# Heritability of head size in a hunted large carnivore, the brown bear (*Ursus arctos*)

**DOI:** 10.1111/eva.12786

**Published:** 2019-03-21

**Authors:** Inger Maren Rivrud, Shane C. Frank, Richard Bischof, Atle Mysterud, Sam M. J. G. Steyaert, Anne G. Hertel, Snorre B. Hagen, Hans Geir Eiken, Jon E. Swenson, Andreas Zedrosser

**Affiliations:** ^1^ Department of Biosciences, Centre for Ecological and Evolutionary Synthesis University of Oslo Oslo Norway; ^2^ Department of Natural Sciences and Environmental Health, Faculty of Technology, Natural Sciences and Maritime Sciences University of South‐Eastern Norway Bø i Telemark Norway; ^3^ Faculty of Environmental Sciences and Natural Resource Management Norwegian University of Life Sciences Ås Norway; ^4^ Norwegian Institute of Bioeconomy Research Svanvik Norway; ^5^ Norwegian Institute for Nature Research Trondheim Norway

**Keywords:** animal model, brown bear, evolvability, heritability, large carnivores, MCMCglmm, pedigree, quantitative genetics

## Abstract

Wild animal populations experience selection pressures from both natural and anthropogenic sources. The availability of extensive pedigrees is increasing along with our ability to quantify the heritability and evolvability of phenotypic traits and thus the speed and potential for evolutionary change in wild populations. The environment may also affect gene expressions in individuals, which may in turn affect the potential of phenotypic traits to respond to selection. Knowledge about the relationship between the genetic and environmental components of phenotypic variation is particularly relevant, given ongoing anthropogenically driven global change. Using a quantitative genetic mixed model, we disentangled the genetic and environmental components of phenotypic variance in a large carnivore, the brown bear (*Ursus arctos*). We combined a pedigree covering ~1,500 individual bears over seven generations with location data from 413 bears, as well as data on bear density, habitat characteristics, and climatic conditions. We found a narrow‐sense heritability of 0.24 (95% CrI: 0.06–0.38) for brown bear head size, showing that the trait can respond to selection at a moderate speed. The environment contributed substantially to phenotypic variation, and we partitioned this into birth year (5.9%), nonadditive among‐individual genetic (15.0%), and residual (50.4%) environmental effects. Brown bear head circumference showed an evolvability of 0.2%, which can generate large changes in the trait mean over some hundreds of generations. Our study is among the first to quantify heritability of a trait in a hunted large carnivore population. Such knowledge about the degree to which species experiencing hunting can respond to selection is crucial for conservation and to make informed management decisions. We show that including important environmental variables when analyzing heritability is key to understanding the dynamics of the evolutionary potential of phenotypic traits.

## 1 INTRODUCTION

Many wild animal populations are experiencing increased selection pressures from environmental sources, both natural and anthropogenic (Allendorf & Hard, 2009; Hendry, Farrugia, & Kinnison, 2008; Van de Walle, Pigeon, Zedrosser, Swenson, & Pelletier, 2018). The human global footprint is now so evident that the current time period may be defined as a new era, the Anthropocene (Boutin & Lane, 2014; Crutzen, 2006; Parmesan, 2006; Pelletier & Coltman, 2018; Waters et al., 2016). Human‐driven climate change is now one of the most important drivers of increasing environmental selection pressures (Walther et al., 2002). In addition, humans can be considered “superpredators” (Darimont, Fox, Bryan, & Reimchen, 2015), with the ability to affect the evolutionary dynamics of prey (Allendorf & Hard, 2009; Bischof et al., 2018; Pigeon, Festa‐Bianchet, Coltman, & Pelletier, 2016; Van de Walle et al., 2018), thereby also affecting how animals adapt to local environmental conditions shaped by anthropogenic pressures. Environmental conditions are expected to affect gene expressions and phenotypes, which may in turn affect the evolutionary potential of phenotypic traits (Falconer & Mackay, 1996; Lynch & Walsh, 1998). Increasing our understanding of how wild populations respond to changing environmental conditions is key to making predictions and preventing undesirable consequences, such as altered life history and potential population collapse (Bischof et al., 2018; Darimont et al., 2015).

Extensive pedigrees are becoming increasingly available for wild animal populations (reviewed in Charmantier, Garant, & Kruuk, 2014). By combining pedigrees with statistical tools, such as the animal model (Henderson, 1953,1973; Kruuk, 2004; Lynch & Walsh, 1998), it is possible to quantify the heritability of phenotypic traits and the potential for evolutionary change in wild populations (Kruuk, Slate, & Wilson, 2008; Wilson et al., 2010). Knowledge about the relative influence of environmental and genetic variation on phenotypic expression is crucial to understanding the response to selection of phenotypic traits under recent climate and land‐use changes (Kruuk et al., 2002). Studies measuring the relative role of phenotypic plasticity and heritability of traits in the wild are typically limited to birds (Charmantier et al., 2008; Husby, Hille, & Visser, 2011), fish (Letcher, Coombs, & Nislow, 2011), rodents (Blumstein, Lea, Olson, & Martin, 2010; Taylor et al., 2012), and ungulates (Kruuk et al., 2000; Robinson et al., 2009), and are exceedingly rare in large carnivores (but see Malenfant, Davis, Richardson, Lunn, & Coltman, 2018).

Body size is positively correlated with fitness in many species (Blanckenhorn, 2000; Kingsolver & Pfennig, 2004). The trait is also to some extent heritable (Postma, 2014) and therefore likely to respond to selection. Here, we combine data from a unique long‐term monitoring program of a hunted large carnivore, the brown bear (*Ursus arctos*; 1985–2014) in Scandinavia, with tissue samples from hunted bears, which allowed us to estimate pedigrees and reproductive success of males and females, based on microsatellite analysis. We estimate the heritability and evolvability of head size (defined as head circumference) and attempt to disentangle the genetic and environmental components of phenotypic variance underlying brown bear head circumference. Head circumference is highly correlated with body mass in brown bears (Dahle, Zedrosser, & Swenson, 2006), and is therefore suitable as a proxy for body size, a trait strongly related to fitness in ursids (Derocher & Stirling, 1998; Zedrosser, Bellemain, Taberlet, & Swenson, 2007). With their large home ranges and direct competition with humans, large carnivores are under particularly strong anthropogenic pressure (Bischof et al., 2018; Zedrosser, Steyaert, Gossow, & Swenson, 2011). Many large carnivores, including the brown bear, are also subject to trophy hunting through size selectivity and male‐biased hunting (Wielgus, Morrison, Cooley, & Maletzke, 2013; Wielgus, Sarrazin, Ferriere, & Clobert, 2001). The evolutionary response to these potential sources of phenotypic selection depends on the heritability and evolvability of the phenotypic traits under selection. Quantitative information about the vulnerability of wild populations to anthropogenic pressures is therefore critical for management and conservation.

## 2 MATERIALS AND METHODS

### 2.1 Study area and population

The study area is situated in south‐central Sweden and in parts of neighboring eastern Norway (see Supporting Information, Figure S1). The area covers Dalarna and Gävleborg counties, as well as portions of Jämtland, Värmland, and Västernorrland counties in Sweden and parts of Hedmark County in Norway. Tree cover is dominated by Scots pine (*Pinus sylvestris*), Norway spruce (*Picea abies*), and birch (*Betula *spp*.*), with the gently rolling landscape consisting mainly of managed forests ranging from clear‐cuts to 100‐year‐old tree stands (Swenson, Jansson, Riig, & Sandegren, 1999), as well as lakes and bogs. It has an inland climate with temperature ranging from an average daily minimum temperature of −7°C in January to maximum 15°C in July (Moe, Kindberg, Jansson, & Swenson, 2007). Snow cover typically lasts from late October to early May, and rainfall averages about 350–450 mm during the snow‐free period (Swenson et al., 1999).

We used data from bears monitored by the Scandinavian Brown Bear Research Project (SBBRP) and dead bears examined by the Swedish State Veterinary Institute (SVA; www.sva.se) from 1985 to 2014. Lone male and female brown bears and females with yearling cubs were immobilized from a helicopter from mid‐April to mid‐May, shortly after den emergence, and fitted with very‐high‐frequency (VHF) collars (from 1985 to 2002; Telonics^®^; Mesa, AZ, USA) with a transition to global positioning system (GPS) collars in later years (from 2003 to 2014; Vectronics Aerospace GmbH, Berlin, Germany). Individuals with VHF collars were tracked and locations recorded with standard triangulation every second week, whereas GPS positioning schedules varied across time and individuals, but fixes were obtained at least once every hour. The position data were compiled in the Wireless Remote Animal Monitoring (Dettki, Brode, Giles, & Hallgren, 2014) database system for validation and management. We removed positions which were not validated or had dilution of precision values >5 (21.7% of all relocations), and retained hourly fixes screened for spatial outliers (<0.1% of the data) following (Bjørneraas, Van Moorter, Rolandsen, & Herfindal, 2010). During capture, sex and size (body weight and head circumference) of all individuals were recorded, and tissue samples were collected for genetic analysis. The ages of bears not first captured as yearlings with their mother were determined by counting the annuli in the root of a rudimentary premolar tooth (Matson et al., 1999). We measured head circumference in cm (at the widest part of the zygomatic arch between eyes and ears) with a tape measure and used it as a surrogate measure of overall size (Bischof, Zedrosser, Brunberg, & Swenson, 2009; Dahle, Zedrosser et al., 2006; Zedrosser, Dahle, & Swenson, 2006). We defined females ≥4 years as adults, that is, the age at which female brown bears can start producing litters in Scandinavia (Zedrosser, Dahle, Støen, & Swenson, 2009). Because all bears were captured within a 2‐week period after den emergence, we did not adjust size for capture date (Zedrosser, Pelletier, Bischof, Festa‐Bianchet, & Swenson, 2013). See Arnemo et al. (2006) and Arnemo, Evans, and Fahlman (2011) for further details on the capture procedures. All capture and handling conformed to the current laws regulating the treatment of animals in Sweden and were approved by the appropriate Swedish ethical committee (Djuretiska nämden i Uppsala).

Bear mortality data are routinely collected by SVA. Swedish regulations require that all bears killed by humans or found dead must be reported to the authorities. Date and location of death, as well as head and other body size measurements, sex, age (based on tooth cementum annuli), and cause of death are recorded when known. Successful hunters are required to provide this information to official inspectors (Bischof, Swenson, Yoccoz, Mysterud, & Gimenez, 2009; Steyaert et al., 2016).

### 2.2 Pedigree

Tissue samples for genetic analysis were stored in 95% EtOH prior to DNA extraction. The amplification and analysis of 16 microsatellites (Frank et al., 2018) were performed following the protocol from Waits, Taberlet, Swenson, Sandegren, and Franzen (2000). Using multiple genotypes belonging to individuals (*N* = 120) captured multiple times and/or also recovered dead, we calculated an error rate from the sum of mismatches between paired loci divided by the total number of loci genotyped.

We used Cervus 3.0 (Kalinowski, Taper, & Marshall, 2007; Marshall, Slate, Kruuk, & Pemberton, 1998) and COLONY (Jones & Wang, 2010) to assign parentage to offspring and build a pedigree (Frank, 2017). In Cervus, we used a critical LOD delta score of 95% against simulations to assign parents, that is, fathers, if mothers were known, or both parents, from a list of candidate parents based on minimum ages of first reproduction (males = 3 years, females = 4 years; Zedrosser et al., 2007; Zedrosser et al., 2009). We then used COLONY for sibship reconstruction, which simultaneously reconstructs unknown father genotypes, enabling us to recover potential fathers and sibship missed in Cervus's parentage assignment (Frank, 2017). Both softwares integrated genotyping error rate into their assignment efforts.

As fitness proxies, we calculated the mean total and yearly number of offspring ± *SD* assigned to each adult male and female. For males, we calculated the mean ± *SD* number of successful breeding attempts each year and the mean ± *SD* number of offspring in each successful breeding attempt. When monitoring data were available, we calculated yearly individual offspring assigned during the monitoring period, that is, from age of first reproduction until the individual was confirmed dead, or telemetry connection was lost.

### 2.3 Home range estimation and environmental covariates

We extracted information on mean bear density, climate, habitat variables, and plant phenology within the individual annual brown bear home ranges from geographic information system (GIS) maps, using R (R Core Team, 2018). Circular annual home ranges were constructed with a fixed sex‐specific radius (18.33 km for males and 8.31 km for females and unweaned males; Dahle & Swenson, 2003), as VHF‐collared individuals often had too few locations to calculate reliable empirical home range estimates. Home range center locations were estimated either as the centroid of the 100% adaptive local convex hull (*a*‐LoCoH) polygon or as the median of all individual relocations, based on a visual inspection of the individual relocations overlaid with the aforementioned centers and corresponding radius. More details on home range estimation can be found in the Supporting Information Appendix S1.

We extracted environmental covariates from the year prior to capture, because body condition after den emergence reflects the previous year's environmental conditions (Zedrosser et al., 2006). If no home range estimate was available from the previous year, we used the home range from the current year. For yearlings, we used their mother's home range from the previous year. As brown bear home range centers are relatively stable after they reach 4 years of age (Støen, Bellemain, Sæbø, & Swenson, 2005; Støen, Zedrosser, Sæbø, & Swenson, 2006), we filled in gaps in the monitoring time series by using the home range center closest in time when the bear was ≥4 years for covariate extraction (*N* = 579). For subadults, whose home range centers are less stable (Dahle, Støen, & Swenson, 2006), gaps in the monitoring time series were filled only if home range centers were (a) available for at least 2 years between 0 and 3 years of age, and (b) the distance between these home ranges was smaller than the mean sex‐specific home range radius, which indicated that the individuals (*N* = 4) had remained in the same area.

The bear density index (number of bears/1,000 km^2^) was extracted from annual raster grids developed using information from bear genetics from scat collection efforts and the Swedish Large Carnivore Observation Index (LCOI), both of which were collected during the fall hunting season (Kindberg, Ericsson, & Swenson, 2009; Kindberg et al., 2011; see Supporting Information Appendix S1 for details). Climate data were obtained from the Swedish Meteorological and Hydrological Institute. These were further downscaled and processed (see Bischof et al., 2018 and Supporting Information Appendix S1), and we derived 21 climate variables that could potentially affect brown bear head circumference. Due to correlations, we conducted a principal component analysis (PCA) to identify patterns of variation (Supporting Information Figure S2). Based on the PCA, 3 climate variables were retained: winter severity (number of days with minimum temperature <−10°C between 1 November year *t* and 30 April year *t* + 1), minimum temperature in May (°C), and mean daily precipitation in the growing season (mm; defined from last day of snow depth >0 cm to first day of minimum temperature <−1°C; Rixen, Dawes, Wipf, & Hagedorn, 2012).

Data on plant phenology were quantified using the satellite‐derived normalized difference vegetation index (NDVI; Pettorelli et al., 2005). NDVI images provide a measure of the vegetation greenness of a given pixel (8 × 8 km) at a given time. As the brown bear is an omnivore, this index can be used as a proxy for forage availability, both with regard to good conditions for berry production (e.g., spring conditions, i.e., timing and duration of spring green‐up) and ungulate availability (Bojarska & Selva, 2012). Further details on estimation and extraction of plant phenology can be found in the Supporting Information Appendix S1. For each individual home range, we obtained the mean day of onset (green‐up) and end of spring and fall (plant senescence), and calculated the duration of the growing season following Bischof et al. (2012) and Rivrud et al. (2016).

A terrain ruggedness index (TRI) was derived from a digital elevation model (DEM) covering the study area. The DEM was rasterized with a resolution of 50 × 50 m, and the TRI was calculated based on the “terrain” function in the “raster” package (Hijmans & van Etten, 2015) in R. This function calculates the mean of the absolute differences between the elevation value of a focal pixel and the value of its neighboring pixels (Wilson, O'Connell, Brown, Guinan, & Grehan, 2007). Road density (km^2^) was extracted from the Swedish National Road Database (http://www.nvdb.se). We extracted the mean TRI and road density within the individual annual home ranges.

### 2.4 Statistical analyses

We used a quantitative genetic mixed model (animal model) to estimate the quantitative genetic parameters, with variance partitioned into genetic and environmental components (Henderson, 1973; Kruuk, 2004; Wilson et al., 2010). Animal models allow for repeated measures on the individuals and for the inclusion of fixed effects that can explain environmental variation that affects the phenotypic trait mean (Kruuk, 2004; Wilson et al., 2010). The model was fitted with the package “MCMCglmm” (Hadfield, 2010) in R.

The phenotypic trait we investigated was brown bear head size, measured as head circumference. Brown bears exhibit large interannual variations in body mass due to hibernation and hypo‐ and hyperphagia (Swenson, Adamič, Huber, & Stokke, 2007). Head circumference is therefore a better proxy for body size than body mass in hibernating species, because its smaller fat deposits make it less sensitive to annual fluctuations in food availability (Derocher & Stirling, 1998; Hertel et al., 2018; Zedrosser et al., 2006). To ensure that head circumference measures were comparable between individuals and years, we used only spring measures taken after den exit (usually the middle or end of April) until May 15.

In addition to the animal identity term needed to estimate the additive genetic variance (*V*_A_), the random effects included mother id to estimate maternal effects (*V*_M_), birth year for natal cohort variance (*V*_BY_), and individual id for repeated measures (nonadditive among‐individual genetic variance *V*_I_). The sum of these, including the residual (or environmental) variance (*V*_R_), constitutes the total phenotypic variance (*V*_P_; Falconer & Mackay, 1996). Heritability (narrow‐sense) was calculated as *h* ^2^ *= V*_A_ */V*_P_ (Falconer & Mackay, 1996), and we also estimated heritability corrected for variation in fixed effects (*V*_F_; de Villemereuil, Morrissey, Nakagawa, & Schielzeth, 2018; Wilson, 2008). To complement the heritability estimates and provide a more general estimate of the evolutionary potential of brown bear head circumference, we estimated the trait evolvability (*I*_A_; Hansen, Pélabon, & Houle, 2011; Houle, 1992). Evolvability was calculated as *I*_A_ = *V*_A_/*m* ^2^, where *m* is the trait mean (Hansen et al., 2011).

Fixed effects included in the model were the environmental covariates bear density, terrain ruggedness, road density, minimum temperature in May, winter severity, mean precipitation, and length of spring. We also added sex to correct for sexual dimorphism and age to correct for head circumference being measured at different ages (categorical with individual categories from 1 to 10 years, and grouped at 11–15, 16–20, and >21 years), as well as the interaction between age and sex. For the fixed effects, we used the default normal priors (Hadfield, 2010). The random effects were assigned parameter‐expanded priors with a mean of zero (alpha.mu = 0) and a scale of 1,000 (alpha.V = 1,000). These priors follow scaled noncentral F‐distributions (V = 1 and nu = 1; Gelman, 2006; Hadfield, 2010). The model was run for 1,500,000 iterations, with a burn‐in of 100,000 and a thinning interval of 300 (*N*_obs_ = 954 and *N*_ind_ = 413). Model diagnostics showed that the chains of the model mixed well with no signs of trends, that a sufficient number of iterations were used, and that there was low autocorrelation among iterations (<0.05).

## 3 RESULTS

### 3.1 Pedigree

The pedigree contained 1,524 individuals, of which 413 were VHF/GPS‐collared individuals used in our analyses. The genotyping error rate was 0.1%, which was calculated from a subset of individuals that had been genotyped at least twice (*N* = 120). Of the marked individuals, 334 (80.9%) and 333 (80.6%) were assigned mothers and fathers, respectively, at a 95% population‐wide confidence level, creating a maternal‐to‐paternal link factor of 1.003. We succeeded in assigning both a mother and a father to 316 individuals (76.5%). Mothers had a mean of 4.6 ± 4.3 offspring assigned in the pedigree (pedigree lifetime reproductive success over the monitoring period ranged from 1 to 22), and fathers were assigned 2.9 ± 2.4 offspring (pedigree lifetime reproductive success over the monitoring period ranged from 1 to 11). Number of offspring ranged from 1 to 4 per litter for mothers (yearly mean 1.86 ± 0.75; note that females typically reproduce every 2–3 years) and 1 to 6 per year for fathers (mean 1.69 ± 0.98). Females annually produced young with on average 1.3 ± 0.5 males (range 1–3), and males with on average 1.09 ± 0.29 females (range 1–2).

### 3.2 Animal model

**Figure 1:**
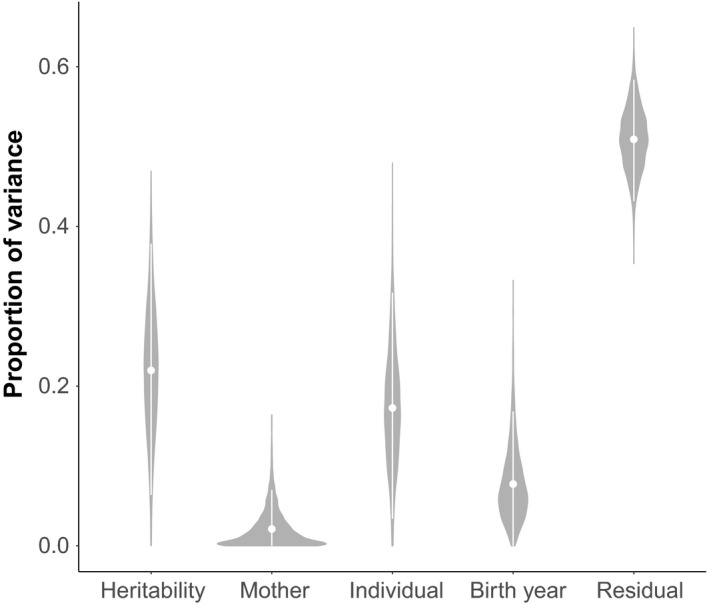
The proportion of variance accounted for by the variance components additive genetic variance (heritability; *V*_A_), maternal variance (*V*_M_), nonadditive among‐individual genetic variance (*V*_I_), birth year variance (*V*_BY_), and environmental variance (residual; *V*_R_). The violins show the empirical posterior distribution of the different components with the corresponding posterior means (white dots) and 95% credible intervals (white bars). The estimates are based on an animal model exploring brown bear head circumference with *N*_obs_ = 954, *N*_ind_ = 413

The narrow‐sense heritability estimate of brown bear head circumference was 0.24 (posterior mode; 95% credible interval (CrI): 0.06–0.38; Table 1 and Figure 1). Birth year accounted for 5.9% of the phenotypic variance (posterior mode = 0.06; 95% CrI: 0–0.17), and nonadditive among‐individual genetic effects accounted for 15.0% of the phenotypic variance (posterior mode = 0.15; 95% CrI: 0.03–0.32). The maternal effects variance converged on zero (posterior mode = 0.0003; 95% CrI: 0–0.07), possibly due to too low sample size, as the mother was not known for all individuals (*N*_obs_ with known mothers = 745, *N*_ind_ = 334). Moreover, the resulting maternal effects estimate may also be due to the model having difficulties separating the birth year variance from the maternal effects variance. Twenty offspring (4.8%) were sole offspring assigned to their respective mothers, and 32 mothers (42.1%) were assigned offspring in 1 year alone. As the percentage of the latter is high, this may affect how well the model can separate maternal effects from birth year effects, and therefore cause one of the variances to converge on zero. Residual, or environmental, variance accounted for 50.4% (posterior mode = 0.50; 95% CrI: 0.43–0.89; Figure 1). We found a repeatability of brown bear head circumference of 0.47 (95% CrI: 0.39–0.56), which can be viewed as an upper limit of heritability. Heritability and repeatability corrected for fixed‐effects variance was 0.02 (posterior mode; 95% CrI: 0.004–0.03) and 0.04 (95% CrI: 0.03–0.05), respectively. The evolvability of brown bear head circumference was 0.002 (posterior mode; 95% CrI: 0.0004–0.003).

**Table 1:** Results from the animal model exploring brown bear head circumference

	Posterior mean	Lower 95% CrI	Upper 95% CrI
Variance components			
*V*_A_	3.02	0.58	5.30
*V*_M_	0.29	<0.001	0.99
*V*_BY_	1.08	0.001	2.44
*V*_I_	2.33	0.49	4.23
*V*_R_	6.91	6.17	7.73
Fixed effects			
Intercept	39.73	37.14	42.06
Age 2	7.42	6.28	8.55
Age 3	11.69	10.65	12.71
Age 4	16.01	15.13	16.97
Age 5	18.38	17.34	19.52
Age 6	18.91	17.80	19.91
Age 7	20.39	19.20	21.68
Age 8	20.24	19.02	21.38
Age 9	21.37	19.92	22.89
Age 10	21.61	20.21	23.15
Age 11–15	22.37	21.37	23.40
Age 16–20	23.57	22.15	24.95
Age 20+	25.73	23.21	28.37
Sex: Male	0.82	0.02	1.65
Age 2 × Sex: Male	−0.46	−1.95	1.06
Age 3 × Sex: Male	2.11	0.65	3.63
Age 4 × Sex: Male	5.69	4.20	7.17
Age 5 × Sex: Male	7.52	5.86	9.16
Age 6 × Sex: Male	10.36	8.70	12.14
Age 7 × Sex: Male	11.71	9.78	13.68
Age 8 × Sex: Male	12.35	10.30	14.39
Age 9 × Sex: Male	13.17	10.88	15.29
Age 10 × Sex: Male	12.28	10.18	14.48
Age 11–15 × Sex: Male	13.75	12.07	15.31
Age 16–20 × Sex: Male	15.41	13.43	17.66
Age 20+ × Sex: Male	11.38	7.33	15.40
Bear density	−2.67	−4.55	−0.57
Road density	1.22	0.12	2.47
Terrain ruggedness	0.09	−0.32	0.52
Mean precipitation	−0.31	−1.44	0.82
Winter severity index	−0.01	−0.02	0.001
Length of spring	0.01	−0.01	0.02
Min. temperature in May	0.09	−0.04	0.24

Note

Estimates of the variance components additive genetic variance (*V*_A_), maternal variance (*V*_M_), birth year variance (*V*_BY_), nonadditive among‐individual genetic variance (*V*_I_), and environmental variance (residual; *V*_R_), and estimates of the fixed effects (beta; posterior mean) are given with their 95% credible intervals (CrI). The reference level for the fixed‐effect “sex” is female. *N*_obs_ = 954, *N*_ind_ = 413.

**Figure 2:**
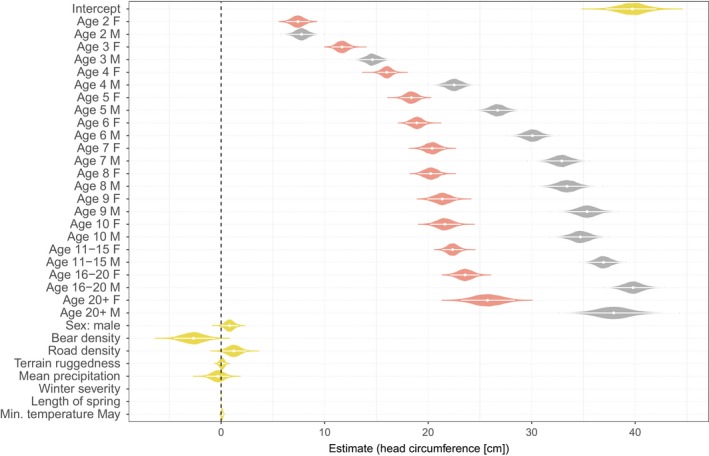
Posterior means (white dots) and their 95% credible intervals (white bars) of the fixed effects in the animal model exploring brown bear head circumference. The violins show the posterior distribution of the different components, and the size is scaled with the same maximum width for visualization. Estimates for female age classes are shown in pink, and for males in gray. Male age class estimates are combined with the intercept to allow for comparison of estimates between the sexes. Note that the estimates for winter severity index and length of spring are shown, but are very small. *N*_obs_ = 954, *N*_ind_ = 413

Bear density showed a significant negative relationship with head circumference, with a 0.27 cm decrease in head circumference when bear density increased by 1 bear/1,000 km^2^ (Table 1 and Figure 2). Head circumference also increased with increasing road density (0.12 cm increase when road density increased by 0.1 km^2^) within the home range and showed a decreasing trend with increasing winter severity (−0.11 cm when the winter severity index increased by 10, but 95% CrI showing a small overlap with 0; Table 1 and Figure 2). As expected, males were overall larger and had a faster growth rate than females. Head circumference increased with age until leveling off around 5 and 7 years of age for females and males, respectively (Table 1 and Figure 2). All remaining fixed‐effect estimates overlapped zero (95% CrI; Table 1 and Figure 2).

## 4 DISCUSSION

With the extensive anthropogenic pressures currently exerted on wild carnivore populations, a quantification of the heritability and evolvability of morphological traits, and thus the speed and potential for phenotypic traits to respond to evolution, is particularly important. Our study provides the first estimate of heritability for brown bear head circumference, a good proxy for body size, with a narrow‐sense heritability of 0.24 (95% CrI: 0.06–0.38). This estimate falls within the range commonly observed for morphological traits in wild animal populations (Kruuk et al., 2002; Teplitsky, Mills, Alho, Yarrall, & Merilä, 2008, and see Postma, 2014 for an overview of publications). Further, we estimated an evolvability of brown bear head circumference of 0.002 (95% CrI: 0.0004–0.003). As brown bear head circumference is heritable, and shows evolvability, consistent directional selection on this trait should lead to a change in the mean of its distribution, if other conditions remain constant and selection on genetically correlated traits is not counteracting the response to selection (Endler, 1986; Falconer & Mackay, 1996; Fisher, 1958). However, this change in trait distribution is rarely observed in wild populations (Coltman et al., 2003), where both heritability and directional selection are often identified (Brookfield, 2016; Kruuk et al., 2002, but see Bonnet, Wandeler, Camenisch, & Postma, 2017). Knowledge about the genetic and environmental components underlying phenotypic traits, and the evolvability of these traits, is therefore crucial to make predictions about how and how fast selection pressures will affect the future distribution of the trait in populations of wild animals. Environmental sources contributed substantially to phenotypic variance in head circumference, with nonadditive among‐individual genetic variance showing 15.0% contribution to phenotypic variance, birth year effects accounted for 5.9% of the phenotypic variance, whereas residual variance, hereunder environmental variance not corrected for in the fixed or random effects, accounted for 50.4%. In our study, the environmental contribution to brown bear head circumference was substantial, suggesting that bears are plastic in relation to environmental impacts (Merilä, 2012). Thus, changing environmental conditions, such as climate change, anthropogenic influences affecting food availability, and hibernation conditions, can mask potential genetic effects on variation in brown bear head circumference, in addition to causing evolutionary change. This creates challenges when making predictions about future trait development in populations experiencing increasing anthropogenic pressures.

Birth year (*V*_BY_) effects on body size are well documented in mammals, including brown bears (Hertel et al., 2018; Zedrosser et al., 2013). Bear body mass varies over the course of the year with bears losing 30%–60% of their autumn body mass during winter hibernation (Swenson et al., 2007). In Sweden, autumn body mass is directly linked to autumn fruit production, in particular bilberry (*Vaccinium myrtillus*; Hertel et al., 2018). The age group most vulnerable to fluctuations in fruit production is cubs of the year, as their first hibernation depends on fat and lean mass reserves acquired through milk from the mother. Over a 10‐year study period, yearling spring body mass was on average 4 kg or 21% lower in the year of lowest as compared to the year of highest bilberry production (Hertel et al., 2018). Although yearling mass does not affect juvenile survival for females in our study population, it is positively associated with measures of female lifetime reproductive success (Zedrosser et al., 2013). Birth year effects explained 5.9% of adult head circumference variation in brown bears. Although many of the fixed effects in the model were explained by variation in autumn food abundance, the effect of birth year likely also captures some of this variation. For males in this population, birth year effects on mass could influence their ability to gain competitive access to breeding females and therefore affect reproductive success (Zedrosser et al., 2007).

Brown bears exhibit considerable variation in body size, both within and between populations (Swenson et al., 2007; Zedrosser et al., 2006, 2011). Habitat productivity and food abundance are important determinants for explaining geographic variation in body size among brown bear populations (McDonough & Christ, 2012; Schwartz, Miller, & Haroldson, 2003), whereas variation within populations reflects niche utilization (Hilderbrand et al., 2018) and/or density dependence (Zedrosser et al., 2006). NDVI‐based metrics for vegetation phenology and climate variables are informative proxies for habitat quality or productivity in wildlife research (Bojarska & Selva, 2012; Pettorelli et al., 2005), and can explain variation in animal performance and life history (Pettorelli et al., 2006; Tafani, Cohas, Bonenfant, Gaillard, & Allainé, 2013). NDVI‐derived metrics did not explain variation in head circumference in our study population. During the fall, the main source for weight gain in brown bears is berries. Hertel et al. (2016) did not find NDVI to be a good predictor of berry production, which may explain the lack of an effect on head circumference in our study. Increased winter severity, that is, colder conditions before and during denning, had a negative effect on brown bear head circumference. This is as expected in a hibernating species, as decreased temperatures are correlated with longer denning periods (Miller, 1990). Winter severity has also been found to affect foraging strategies in brown bears (Bojarska & Selva, 2012), which can influence energy intake and body growth. As expected, head circumference in our study population was negatively affected by brown bear density (Zedrosser et al., 2006). Density‐dependent effects, such as competition for food among individuals, result in decreased body size, as have been documented in a range of species (Fowler, 1987), including brown bears (Zedrosser et al., 2006).

Individual home range composition of bears often differs in terms of habitat availability and anthropogenic features, like roads. Our results suggest a positive effect of road density on head circumference, which may appear counterintuitive. However, our study area is characterized by a very high density of logging roads (1 ± 0.5 km/km^2^, ranging between 0 and 4.6 km/km^2^; Ordiz, Kindberg, Sæbø, Swenson, & Støen, 2014). The only virtually roadless terrain in our study area consists of large boggy areas, which are of little interest for forestry, because of their low primary production. These areas are also poor in terms of food availability, like berries (Hertel et al., 2016). In brown bears, adult males often shape the socio‐spatial structure of the population, due to their despotic nature (Elfström, Zedrosser, Jerina et al., 2014; Rode, Farley, & Robbins, 2006; Steyaert, Kindberg, Swenson, & Zedrosser, 2013). This implies that less competitive (i.e., smaller) individuals can be pushed into less favorable habitat, such as habitats poor in food resources (Ben‐David, Titus, & Beier, 2004; Steyaert, Reusch et al., 2013) or relatively close to people (Elfström, Zedrosser, Støen, & Swenson, 2014; Nellemann et al., 2007). Therefore, we suggest that areas with low road density can reflect poor habitat quality, which may be more often occupied by smaller individuals.

The animal model presented here includes, in addition to the variance components, several fixed effects. The inclusion of environmental factors as fixed effects in these models is common, to account for as much environmental variation as possible when estimating phenotypic variance (Kruuk, 2004; Wilson et al., 2010). However, the inclusion of fixed effects influences the heritability estimate, as *h* ^2^ is calculated conditionally on the particular set of environmental components in a given model (de Villemereuil et al., 2018; Wilson, 2008). Our study shows a narrow‐sense heritability of 0.24 calculated as *V*_A_/*V*_P_. As expected, by correcting for the fixed‐effect variance (*V*_F_) when estimating heritability, the heritability estimate became considerably lower (posterior mode: 0.02; 95% CrI: 0.004–0.03; de Villemereuil et al., 2018). Both heritability estimates reported here are useful for inference regarding the speed of response to selection, but neither is optimal for comparison across studies, traits, or species. Evolvability, measured as additive genetic variation that is scaled by the square of the trait mean, provides a measure of the percent expected change in a trait under a given unit of selection strength (Hansen et al., 2011; Houle, 1992). Thus, evolvability allows for a direct measure of the evolutionary potential of a given trait, which is also comparable across traits and species. Here, we found an evolvability of 0.002, which suggests that brown bear head circumference is expected to change by 0.2% per generation per unit selection. This rate is within the range reported for morphological traits in Hansen et al. (2011) and can lead to large changes over just a few hundred generations.

We used a pedigree covering ~1,500 individuals from a population of brown bears facing potential selection pressures from anthropogenic sources, such as hunting and climate change. This allowed us to identify moderate heritability of head circumference. Body size is a trait often targeted by trophy hunting (Wielgus et al., 2013). Even though brown bear head circumference has adaptive potential, there was also a substantial amount of variation attributed to residual, or environmental variance (*V*_R_; 50.4%). Thus, consistent and strong selection pressures on brown bear body size are likely necessary for adaptation to occur rapidly. Such selection pressures are common under heavy trophy hunting, and have led to an evolutionary response in species such as bighorn sheep (*Ovis canadensis*), where trophy size declined under intense trophy hunting (Pigeon et al., 2016). Moreover, evolutionary recovery in horn size was not detected after the hunting pressure was reduced, indicating that selection pressures from trophy hunting can be stronger than natural selection pressures (Allendorf & Hard, 2009; Pigeon et al., 2016). Although trophy hunting is uncommon in our study population and there is little size‐bias between the sexes in hunting records (Bischof, Swenson et al., 2009), studies have found some indications of size selectivity. Leclerc, Walle, Zedrosser, Swenson, and Pelletier (2016) showed that adult female and yearling brown bears shot by hunters were relatively larger than records from captured individuals in Sweden. Also, Zedrosser et al. (2013) found a positive effect of yearling mass (correlated with body size later in life) on lifetime reproductive success, as well as individual fitness. Similarly, Bischof et al. (2018) found a significant positive effect of head circumference as yearling (as a proxy of body size later in life) on reproduction, where larger individuals had a higher probability of producing a litter any given year, and survival, where larger individuals had a higher probability of being killed by hunters. In other brown bear systems, trophy hunting is more common, leading to male‐biased harvest and potential size selectivity (Miller, Schoen, & Schwartz, 2017; Wielgus et al., 2013). Knowledge about the heritability and evolvability of targeted traits and the evolutionary potential and speed of response to selection, and about the degree of trait plasticity, is therefore crucial to make informed management decisions and ensure conservation of hunted populations.

## CONFLICT OF INTEREST

None declared.

## AUTHOR CONTRIBUTIONS

IMR, AZ, JES, and AM planned the study. JES, AZ, SCF, RB, AGH, and SMJGS collected field data. IMR, SCF, RB, AGH, SMJGS, and AZ extracted and formatted the covariates. SCF, SBH, and HGE developed the pedigree. IMR developed the model and wrote the first draft of the manuscript. All authors contributed to subsequent versions.

## Data Availability

The data set and pedigree have been deposited in the online repository of the University of South‐Eastern Norway: https://doi.org/10.23642/usn.7770785.v1.
