# Supplementary material for: Heritability of head size in a hunted large carnivore, the brown bear (Ursus arctos)
Source: Evol Appl. 2019 Mar 21;12(6):1124–35. doi: 10.1111/eva.12786 (PMC6597896; doi:10.1111/eva.12786)
Supplement: Supplementary file 1 [file EVA-12-1124-s001.docx]

# Supporting information for

## Heritability of head size in a hunted large carnivore, the brown bear

## (*Ursus arctos*)

IM Rivrud, SC Frank, R Bischof, A Mysterud, SMJG Steyaert, AG Hertel, SB Hagen, HG Eiken, JE Swenson and A Zedrosser

Published in Evolutionary Applications

## Study area


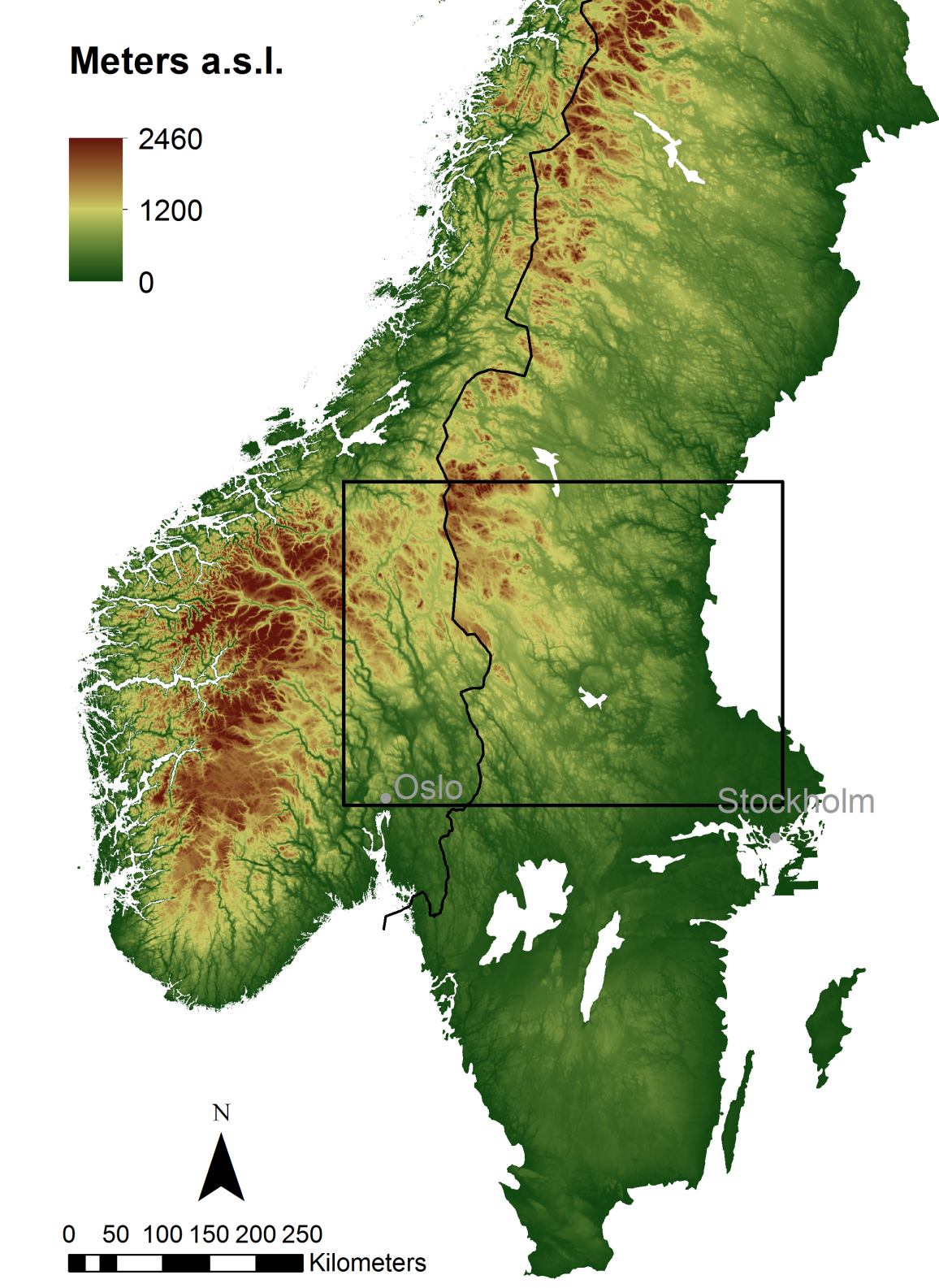


**Figure S1.** Map of the study area (black rectangle) situated in southern Sweden and parts of Norway.

## Home range estimation

Circular individual annual home range centers with a fixed sex-specific radius ([18.33 km for males and 8.31 km for females and unweaned males (family groups); Dahle & Swenson, 2003](#_ENREF_5)) were constructed to extract individual-based environmental covariates. We used centers and corresponding radii, as many VHF/GPS-collared individuals had too few observations to reliably construct empirical home ranges. For all individuals, we calculated the median of all relocations as the home range center, and compared this to the centroid of the home range polygon when possible. For comparison with the median home range center estimates, we estimated home range centers by calculating the 100% adaptive Local Convex Hull (*a*-LoCoH) polygon. *a*-LoCoH home ranges were estimated using the “adehabitatHR” package in R ([Calenge, 2006](#_ENREF_4); [R Core Team, 2018](#_ENREF_14)); a minimum of 15 annual relocations per individual were required. The *a*-value was chosen based on Getz et al. ([2007](#_ENREF_8)), who stated that an *a*-value larger than the two longest distances between relocations should always yield the 100% density isopleth. We used this value when possible, and increased it by adding the third, fourth, or fifth largest distance if the home range estimate failed with a lower *a*-value. After calculating the median and *a*-LoCoH home range center, we added the corresponding radius to the centers and conducted a visual inspection of the home range estimates overlaying the actual relocations. We chose the home range center that best represented the individual relocation pattern, typically the *a*-LoCoH center unless there were few observations. If a monitored bear was recovered as dead, and VHF or GPS locations were not available, the death location was used as the home range center for that year.

## Environmental covariates

### Bear density

The annual bear density index was estimated using two sources of information: (1) bear genetics from scat collection efforts and (2) the Swedish Large Carnivore Observation Index (LCOI), both of which were collected during the fall hunting season ([Kindberg, Ericsson, & Swenson, 2009](#_ENREF_11); [Kindberg et al., 2011](#_ENREF_12)). This information came from Swedish hunters, whose efforts covered virtually the entire area of the four counties of Gävleborg, Dalarna, Jämtland, and Västernorrland ([for more details on collection efforts, see Kindberg et al., 2009](#_ENREF_11)). For each county, scat collection data were used to create spatially explicit density index distributions, according to the year in which scat collection took place. We adopted Jerina et al.'s ([2013](#_ENREF_10)) method in summing the weighted values of individual bear scat locations on a 10 x 10 km cell-sized grid, in order to account for the different number of samples among individuals. Grid cells were then smoothed with a 3 x 3 filter to derive county-specific density index distributions. Year of scat collection varied among counties. Thus, because these maps were not directly comparable without a temporal correction, we used the LCOI. The Swedish LCOI, which represented the relative change of bear abundance over time, was initiated in 1998 by the Swedish Association for Hunting and Wildlife Management ([Kindberg et al., 2011](#_ENREF_12)). Moose (*Alces alces*) hunters report the number of bears observed during the first week of hunting, and the index is effort-corrected using hunter-hours ([Ericsson & Wallin, 1999](#_ENREF_6); [Sylvén, 2000](#_ENREF_17)). We approximated temporal trends in the LCOI for the period 1998-2015 for each county by fitting quadratic models using LCOI as the response and year and year-squared as predictors ([for details see Frank et al., 2018](#_ENREF_7)). The annual density was resampled to raster maps with a 1 x 1 km resolution. The mean bear density index was extracted for the respective years for each individual home range.

### Climate variables

Climate variables were obtained by downscaling and interpolating data from weather stations within the study area (provided by the Swedish Meteorological and Hydrological Institute), with 5-km resolution ([see Bischof et al., 2018 for details](#_ENREF_2)). Initially, we obtained a range of climate variables that could possibly affect brown bear head circumference, either directly, or indirectly through effects on e.g. foraging or denning. To avoid correlations and reduce the complexity of the final model, we carried out a principal component analysis (PCA) including 21 climatic variables to identify patterns of variation in the data. The input data in the PCA was 10,000 random locations from a 1984-2014 climate grid time series, sampled from the same date and location for each variable. Based on this PCA (Supporting Information Fig. S2), we retained 3 climate variables for the final analysis (winter severity [number of days with minimum temperature < -10°C between 1 November year t and 30 April year t+1], minimum temperature in May (ºC), and mean daily precipitation in the growth season [mm; defined from last day of snow depth > 0 cm to first day of minimum temperature < -1ºC]) ([Rixen, Dawes, Wipf, & Hagedorn, 2012](#_ENREF_16)).

### Plant phenology

Time series of NDVI imagery can be used to estimate plant phenology, from which the onset, end, and length of the spring or the growing season can be extracted ([Hird & McDermid, 2009](#_ENREF_9)). Images were downloaded from the NOAA AVHRR GIMMS data set available online via FTP server of the NASA Ames Ecological Forecasting Lab (http://ecocast.arc.nasa.goc/data/pub/gimms/3g.v0). We used the package “gimms” ([Pinzon & Tucker, 2014](#_ENREF_13)) in R to download and rasterize the NDVI images and corresponding images of pixel quality values. The satellite images were available through this package as semimonthly (16-day) images from 1981-2013, with a pixel resolution of 8 x 8 km, and NDVI values were scaled between 0 and 1.

In the resulting NDVI rasters, we identified the pixels covered by the bear home ranges and constructed annual NDVI time series based on the 16-day images. The annual NDVI time series was subjected to a series of processing steps, including filtering according to pixel quality, noise reduction with a 3 x 3 median filter ([Bischof et al., 2012](#_ENREF_3); [Hird & McDermid, 2009](#_ENREF_9)), and smoothing. We kept only pixel qualities of 1 and 2 (good value), as well as values 4 and 6, which are most likely snow values ([Pinzon & Tucker, 2014](#_ENREF_13)). The latter were retained and set to 0, to avoid gaps in the NDVI time series, which may cause problems when smoothing. For smoothing, we fitted a double-logistic function to the annual filtered and scaled NDVI time series, according to Beck et al. ([2006](#_ENREF_1)) and Bischof et al. ([2012](#_ENREF_3)) and adapted by Rivrud et al. ([2016](#_ENREF_15)). From this function, we extracted parameters for each individual home range, describing the day of onset and end of spring (green-up) and fall (plant senescence), as well as the duration of the seasons ([see Rivrud et al., 2016 for details](#_ENREF_15)).

##
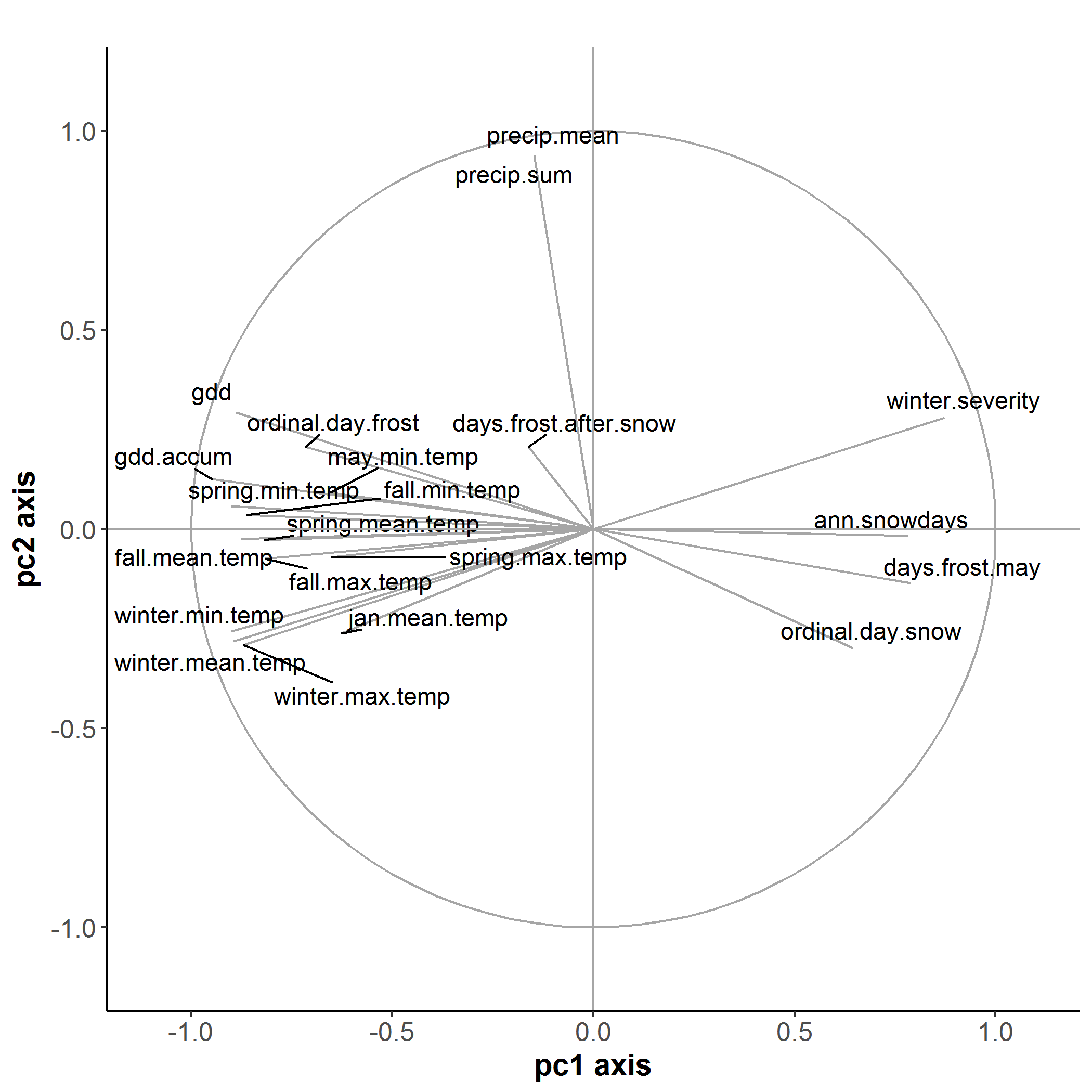


## Figure S2. Principal component analysis (PCA) of the climate data for the study area in southern Sweden—southeastern Norway. Accum = accumulated, ann = annual, gdd = growing degree days, jan = January, precip = precipitation, temp = temperature.

## Literature cited

Beck, P. S., Atzberger, C., Høgda, K. A., Johansen, B., & Skidmore, A. K. (2006). Improved monitoring of vegetation dynamics at very high latitudes: A new method using MODIS NDVI. *Remote Sensing of Environment*, *100*, 321-334.

Bischof, R., Bonenfant, C., Rivrud, I. M., Zedrosser, A., Friebe, A., Coulson, T., . . . Swenson, J. E. (2018). Regulated hunting causes life history makeover in bears. *Nature Ecology & Evolution*, *2*, 116-123.

Bischof, R., Loe, L. E., Meisingset, E. L., Zimmermann, B., Van Moorter, B., & Mysterud, A. (2012). A migratory northern ungulate in the pursuit of spring: Jumping or surfing the green wave? *American Naturalist*, *180*, 407-424.

Calenge, C. (2006). The package "adehabitat" for the R software: A tool for the analysis of space and habitat use by animals. *Ecological Modelling*, *197*, 516-519.

Dahle, B., & Swenson, J. E. (2003). Home ranges in adult Scandinavian brown bears (*Ursus arctos*): effect of mass, sex, reproductive category, population density and habitat type. *Journal of Zoology*, *260*, 329-335.

Ericsson, G., & Wallin, K. (1999). Hunter observations as an index of moose *Alces alces* population parameters. *Wildlife Biology*, *5*, 177-185.

Frank, S. C., Leclerc, M., Pelletier, F., Rosell, F., Swenson, J., Bischof, R., . . . Zedrosser, A. (2018). Sociodemographic factors modulate the spatial response of brown bears to vacancies created by hunting. *Journal of Animal Ecology*, *87*, 247-258.

Getz, W. M., Fortmann-Roe, S., Cross, P. C., Lyons, A. J., Ryan, S. J., & Wilmers , C. C. (2007). LoCoH: Nonparameteric kernel methods for constructing home ranges and utilization distributions. *PLoS ONE*, *2*, e207.

Hird, J. N., & McDermid, G. J. (2009). Noise reduction of NDVI time series: An empirical comparison of selected techniques. *Remote Sensing of Environment*, *113*, 248-258.

Jerina, K., Jonozovič, M., Krofel, M., & Skrbinšek, T. (2013). Range and local population densities of brown bear *Ursus arctos* in Slovenia. *European Journal of Wildlife Research*, *59*, 459-467.

Kindberg, J., Ericsson, G., & Swenson, J. E. (2009). Monitoring rare or elusive large mammals using effort-corrected voluntary observers. *Biological Conservation*, *142*, 159-165.

Kindberg, J., Swenson, J. E., Ericsson, G., Bellemain, E., Miquel, C., & Taberlet, P. (2011). Estimating population size and trends of the Swedish brown bear *Ursus arctos* population. *Wildlife Biology*, *17*, 114-123.

Pinzon, J., & Tucker, C. (2014). A Non-Stationary 1981-2012 AVHRR NDVI3g Time Series. *6*, 6929-6960.

R Core Team. (2018). *R: A language and environment for statistical computing* (Vol. https://www.R-project.org/). Vienna, Austria: R Foundation for Statistical Computing.

Rivrud, I. M., Bischof, R., Meisingset, E. L., Zimmermann, B., Loe, L. E., & Mysterud, A. (2016). Leave before it’s too late: Anthropogenic and environmental triggers of autumn migration in a hunted ungulate population. *Ecology*, *97*, 1058-1068.

Rixen, C., Dawes, M. A., Wipf, S., & Hagedorn, F. (2012). Evidence of enhanced freezing damage in treeline plants during six years of CO2 enrichment and soil warming. *Oikos*, *121*, 1532-1543.

Sylvén, S. (2000). Effects of scale on hunter moose *Alces alces* observation rate. *Wildlife Biology*, *6*, 157-165.
